# Impaired hematopoiesis and delayed thrombopoietic recovery following sublethal irradiation in SRC-3 knockout mice

**DOI:** 10.3892/mmr.2014.2043

**Published:** 2014-03-12

**Authors:** J. JIN, Y. WANG, J. WANG, Y. XU, S.L. CHEN, J.P. WANG, Y.P. SU

**Affiliations:** 1 Institute of Combined Injury, State Key Laboratory of Trauma, Burns and Combined Injury, Chongqing Engineering Research Center for Nanomedicine, College of Preventive Medicine, Third Military Medical University, Chongqing 400038, P.R. China; 2 Department of Hematology, Daping Hospital, Third Military Medical University, Chongqing 400042, P.R. China

**Keywords:** steroid receptor coactivator-3, hematopoiesis, irradiation

## Abstract

The objective of the present study was to investigate the role of the steroid receptor coactivator-3 (SRC-3) in hematopoiesis of mouse bone marrow (BM) following total body irradiation (TBI). SRC-3^−/−^ mice and wild-type (WT) mice were exposed to 4.5 Gy γ rays. Immunoblotting analysis revealed that the SRC-3 protein (p160) levels in normal BM-nucleated cells in WT were higher than in SRC-3^−/−^ mice. Furthermore, peripheral blood cell counts, BM cellularity and colony-forming unit (CFU) assays were performed following irradiation. The results showed that peripheral blood cells were significantly lower in number and recovered less rapidly in irradiated SRC-3^−/−^ mice as compared with control animals. BM-nucleated cell and CFU counts were significantly decreased in SRC-3^−/−^ mice on the 7th and 14th day. Of note, the recovery of platelet (PLT) and megakaryocytic lineage were more depressed than the granulocytic and erythroid lineage in SRC-3^−/−^ mice. In conclusion, the present study demonstrated that the hematopoietic ability in SRC-3 knockout mice is severely impaired following a sublethal dose of irradiation.

## Introduction

Steroid receptor coactivator-3 (SRC-3/AIB1/ACTR/pCIP/RAC3/TRAM-1) is a member of the p160 steroid receptor coactivator family. The SRC-3 gene is located on chromosome 20q12-12. The SRC-3 protein is ~160 kDa and contains three basic structural domains, consistent with the other two SRC family members (SRC-1 and SRC-2) (1–5). SRC-3 is able to interact with nuclear receptors and other transcription factors to enhance their effects on target gene transcription (6). Accumulating *ex vivo* studies indicate that SRC-3 has an important role in physiological and pathological functions involved in cell proliferation, cell differentiation, oncogenesis, cancer metastasis, developmental event regulation and physiological processes including somatic growth, sexual maturation, female reproductive function, energy metabolism and the formation of certain tumors (7–10).

Although numerous biological roles of SRC-3 have been identified, its involvement in hematopoiesis remains to be elucidated. Data from *in vitro* studies have revealed that SRC-3 was overexpressed in certain blood cancer cells and were able to affect cell proliferation and anti-apoptosis (11–13). It is suggested that SRC-3 has a role in the hematopoietic system. However, studies on the role of SRC-3 in the hematopoietic system in SRC-3 knockout (SRC-3^−/−^) mice are rare, particularly studies on mice following irradiation. In the present study, using the SRC-3^−/−^ mouse model, it was validated that disruption of SRC-3 in mice was able to impair hematopoiesis and influence hematopoietic recovery following sublethal total body irradiation (TBI).

## Materials and methods

### Animals

**Figure 1:**
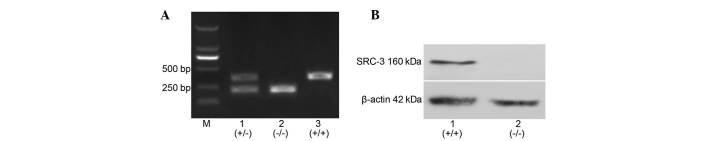
Expression of SRC-3 protein in BM-nucleated murine cells. (A) Confirmation of genotype in SRC-3 mutant mice. Lane M, DNA molecular size marker; lane 1, tail DNA of SRC-3+/− mice with mixture of three primers and bands at 450 and 230 bp indicate the heterozygote; lane 2, tail DNA of SRC-3^−/−^ mice with a mixture of primers 1 and 3, with the 230 bp band indicating the knockout (SRC-3^−/−^) mice; lane 3, tail DNA of SRC-3^+/+^ mice with a mixture of primer 1 plus primer 2, with the 450 bp band indicating wild-type (SRC-3^+/+^) mice. (B) Differential expression of SRC-3 protein in BM-nuclear cells of SRC-3^+/+^ and SRC-3^−/−^ mice. SRC-3, steroid receptor coactivator-3; BM, bone marrow.

SRC-3^−/−^ mice were kindly provided by Professor Jianming Xu (Molecular and Cellular Biology Laboratory, Baylor College of Medicine, Houston, USA). The SRC-3 mutant colony was maintained by interbreeding heterozygous pairs. The mice had a mixed 129/SvEvxC57BL/6J genetic background. Female SRC-3^−/−^ mice and wild-type (WT) counterparts (age, 8–10 weeks) were used in this experiment. Mice were provided with sterilized water and food *ad libitum* in a pathogen-free animal facility. Experimental protocols were approved by the Animal Care Committees of the Third Military Medical University. Genotypes were determined by PCR using tail DNA (Fig. 1A) (7). For PCR analysis, the WT (SRC-3^+/+^) allele was detected using primer 1: 5′-GATGAGTGGACTAGGCGAAAGCTCT-3′ and primer 2: 5′-GCTGAGATTTGCAGAGATGAGCTC-3′. This primer pair amplified a 450-bp fragment from the SRC-3^+/+^ mice. DNA was also amplified using primers 1 and 3: 5′-GGCGATTAAGTTGGGTAACGCCAG-3′, which is located in the LacZ indicator to detect the mutant of the SRC-3 allele. This primer pair amplified a 230-bp fragment from SRC-3^−/−^ mice. The three mixed primers amplified the of 450 and 230 bp fragments to detect the heterozygote.

### Irradiation and mouse treatment

Total body irradiation (TBI) of mice was performed using ^60^Co γ-radiation [4.5 Gy total dose; 0.934 Gy/min at room temperature (25±2°C)]. Mice were divided into the irradiated WT mice (n=16) and irradiated SRC-3^−/−^ mice (n=16). The observation time-points for peripheral blood counts were on days 3, 7, 11, 14, 21 and 28 following TBI. For the mechanistic investigation, every third mouse in each irradiated group was sacrificed on days 7 and 14 following irradiation.

### Peripheral blood hematology

Using a capillary tube, peripheral blood was collected from the tail vein of the mouse and mixed with EDTA in 1.5 ml tubes. Complete blood cell counts were analyzed using a Sysmex 800 i (Sysmex Co. Ltd., Bangkok, Thailand) automated cell counter.

### Bone marrow (BM)-nucleated cell counts

For the preparation of a BM-nucleated cell suspension, mice were sacrificed at the required time-point and BM cells were flushed from femurs with Iscove’s modified Dulbecco’s medium (IMDM; Hyclone, Logan, USA) containing 2% fetal bovine serum (FBS; Gibco, Grand Island, NY, USA). Following lysis of the erythrocytes and two washes with PBS, the single BM-nucleated cells were resuspended. The number of the nucleated cells was determined using a hemocytometer and expressed as total cells (x10^6^)/mice.

### Mouse colony-forming unit (CFU) culture and counts

The CFU assay was performed using a semi-solid culture medium. All the following culture mediums were purchased from Stem Cell Technologies (Vancouver, Canada) and performed according to the manufacturer’s instructions. A single-nucleated cell suspension of mouse BM cells was prepared as described in the aforementioned method, and the density of the suspended cells was adjusted to 2×10^5^/ml for culture. In order to maintain colony-forming unit-erythroids (CFU-Es) and colony-forming unit-granulocytes/macrophages (CFU-GMs), cell suspensions were added to M3334 and M3534 methylcellulose-based media and incubated at 37°C with 5% CO_2_ and ≥95% humidity for three or seven days, respectively. CFU-Es and CFU-GMs were then counted under a light microscope. To maintain colony-forming unit-megakaryocytes (CFU-MKs), the cell suspension was added to MegaCult medium (cat no. 4905) and mixed with cytokines (rmIL-3 [10 ng/ml], rhIL-6 [20 ng/ml], rhTPO [50 ng/ml]) and collagen (cat no. 4902). Following incubation at 37°C with 5% CO_2_ at ≥95% humidity for seven days, cells were stained with acetylthiocholine iodide for 5 h and counterstained with Harris’ hematoxylin solution for 30 sec. All CFU-MK clones, which appeared brown under the light microscope to detect all megakaryocytic progenitors, were counted.

### Histology

Three mice of each strain were sacrificed on the 7th and 14th day following irradiation and biopsies were taken from tibias and stored in buffered formalin. Specimens were embedded in paraffin, sectioned at 5 μm and then stained with hematoxylin and eosin (H&E). The stained slides were studied and the microscopic images were captured using a charge-coupled device (CCD) camera (Olympus, Tokyo, Japan). All the images used in the present study were original and unmodified.

### Immunoblotting

Single-nucleated cell suspensions of mouse BM in PBS were prepared according to the aforementioned method. Proteins were extracted from nucleated cells using protein extraction reagent (Pierce Biotechnology, Inc., Rockford, IL, USA). The protein concentration was determined by the Bradford method. The protein samples (40 μg per lane) were separated by 8% SDS-PAGE and then electroblotted using a PVDF membrane. Following incubation with the primary anti-SRC-3 antibody (Santa Cruz Biotechnology, Santa Cruz, CA, USA) at 4°C overnight and with secondary antibody (Beyotime, Shanghai, China) at 37°C for 2 h, blots were developed by exposure to a medical X-ray film. β-actin was immunoblotted with β-actin antibody (Beyotime) as the control.

### Statistical analysis

Statistical analysis was performed using the SPSS version 11.0 (SPSS, Inc., Chicago, IL, USA). Data were presented as the mean ± standard deviation (SD). A one-way analysis of variance (ANOVA) was adopted to evaluate differences between groups. P<0.05 was considered statistically significant.

## Results

### Expression of SRC-3 protein in murine BM nucleated cells

The SRC-3 protein (160 kDa) was extracted from BM-nucleated cells of WT and SRC-3^−/−^ mice separately and immunoblotted. The results showed that the SRC-3 protein was expressed in BM-nucleated cells of WT mice; however, not in those of SRC-3^−/−^ mice (Fig. 1B).

### Changes in mouse peripheral blood cells following TBI

**Figure 2:**
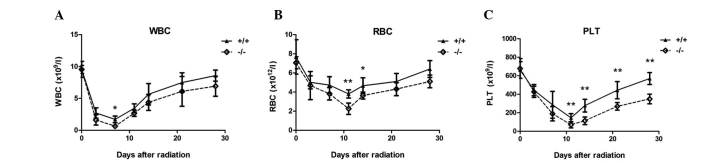
Peripheral blood cell counts in WT and SRC-3^−/−^ mice following 4.5 Gy irradiation shown as the mean ± standard deviation (n=6). (A) WBC counts reached a minimum on day 7, significantly lower in SRC-3^−/−^ mice than in WT mice. (B) RBC counts reached a minimum on day 11, significantly lower in SRC-3^−/−^ mice than in WT mice on days 11 and 14. (C) PLT counts reached a minimum on day 11, significantly lower in SRC-3^−/−^ mice than in WT mice on days 11, 14, 21 and 28. ^*^P<0.05, ^**^P<0.01. WT, wild-type; SRC-3, steroid receptor coactivator-3; WBC, white blood cells; RBC, red blood cells; PLT, platelets.

To determine the hematopoietic damage caused by irradiation, the changes of peripheral blood cells were studied as direct indicators. Changes in white blood cell (WBC), red blood cell (RBC) and platelet (PLT) counts are shown in Fig. 2. Following TBI, counts of circulating WBCs, RBCs and PLTs markedly decreased in WT and SRC-3^−/−^ mice. All peripheral blood cell counts were lower in SRC-3^−/−^ mice than in WT mice at all examined time-points. The minimum WBC, RBC, and PLT counts were all significantly lower in SRC-3^−/−^ mice than in WT mice, respectively (P<0.01). Moreover, the recovery rate of blood cells was different in the two groups: WBCs recovered rapidly in the two groups up to day 28 post-irradiation, with counts almost recovered in WT mice and reaching pre-irradiation values of 81% in SRC-3^−/−^ mice (Fig. 2A). RBCs recovered gradually in the two groups up to day 28 post-irradiation, with counts almost recovered in WT mice and reaching pre-irradiation values of 75% in SRC-3^−/−^ mice (Fig. 2B). PLTs recovered gradually in the two groups up to day 28 post-irradiation. Of note, PLT counts in SRC-3^−/−^ mice were significantly lower on days 11, 14, 21 and 28 than in WT mice and reached pre-irradiation values of 51% (P<0.01) (Fig. 2C).

### Assays performed on BM-nucleated cells following TBI

**Figure 3:**
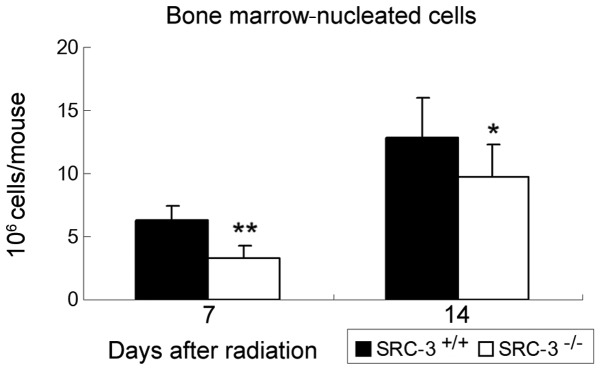
The cellularity of BM-nucleated cells in WT and SRC-3^−/−^ mice on the 7th and 14th day after TBI shown as mean ± standard deviation (n =3). The nucleated cell numbers in SRC-3^−/−^ mice were significantly lower than in WT mice on days 7 and 14. ^*^P<0.05, ^**^P<0.01. BM, bone marrow; WT, wild-type; SRC-3, steroid receptor coactivator-3; TBI, total body irradiation.

**Figure 4:**
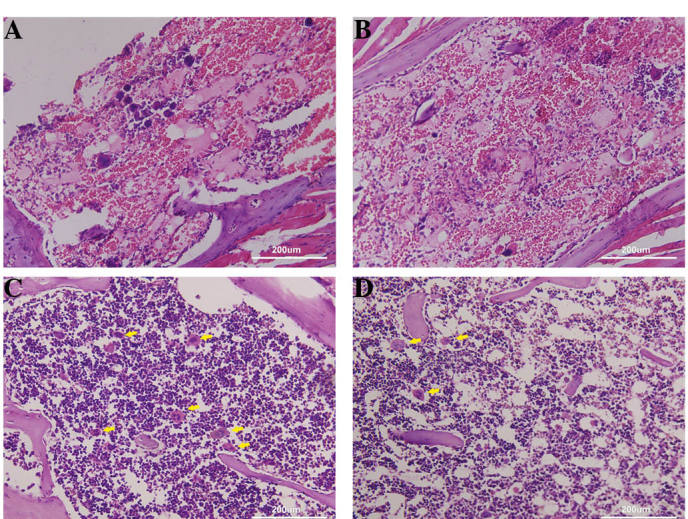
Histological analyses. Irradiated mice (n=3). (A) Irradiated WT mice on the 7th day (B) Irradiated SRC-3^−/−^ mice on the 7th day. (C) Irradiated WT mice on the 14th day (D) Irradiated SRC-3^−/−^ mice on the 14th day (stained with hematoxylin and eosin; magnification, ×200). The arrows show megakaryocytes. WT, wild-type; SRC-3, steroid receptor coactivator-3.

To determine whether the decreased peripheral blood cell counts changed with the reduced cellularity of BM following TBI, the BM-nucleated cells were counted in WT and SRC-3^−/−^ mice on the 7th and 14th day following irradiation. The BM cellularity was reduced in the WT and SRC-3^−/−^ mice. However, the number of BM-nucleated cells in SRC-3^−/−^ mice was significantly lower on the 7th and 14th day following irradiation (P<0.05) (Fig. 3). Histological examination also showed a greater reduction in the number of hematopoietic cells, particularly of megakaryocytes in the BM of SRC-3^−/−^ mice (Fig. 4).

### Assessment of CFU following TBI

**Figure 5:**
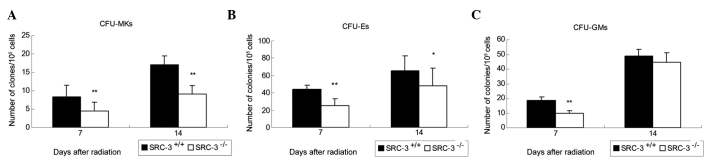
CFU assays in WT and SRC-3^−/−^ mice on the 7th and 14th day after TBI shown as the mean ± standard deviation (n=3). (A) CFU-MK was significantly lower on days 7 and 14 than in WT mice. (B) CFU-E in SRC-3^−/−^ mice was significantly lower on days 7 and 14 than in WT mice. (C) CFU-GMs in SRC-3^−/−^ mice were significantly lower on day 7 than in WT mice. ^*^P<0.05, ^**^P<0.01. CFU, colony-forming units; WT, wild-type; SRC-3, steroid receptor coactivator-3; TBI, total body irradiation; CFU-MKs, colony-forming unit-megakaryocytes; CFU-Es, colony-forming unit-erythroids; CFU-GMs, colony-forming unit-granulocytes/macrophages.

To assess the hematopoietic ability of BM-nucleated cells, the cells were harvested from the BM of SRC-3^−/−^ and WT mice on the 7th and 14th day following TBI and subjected to CFU assays (Fig. 5). On the 7th day, CFU-GM, CFU-E, and CFU-MK counts were significantly lower in SRC-3^−/−^ mice than in WT mice (P<0.01). On the 14th day, CFU-MK and CFU-E counts in SRC-3^−/−^ mice remained significantly lower than in WT mice (P<0.01, P<0.05, respectively). CFU-GM counts in SRC-3^−/−^ mice were also lower; however, there was no statistical significance.

## Discussion

The present study demonstrated the marked differences in the effect of TBI on hematopoietic cells between WT and SRC-3^−/−^ mice. Effects on peripheral blood cells, changes in BM-nucleated cells and colony-forming units of hematopoietic cells in WT and SRC-3^−/−^ mice were examined. A notable impaired hematopoiesis was caused by a sublethal dose of irradiation in SRC-3^−/−^ mice.

The radiation-induced damage is generally considered to result primarily from the effects of radiation on hematopoietic cells. In the present study, the number of peripheral blood cells was significantly lower and the hematopoietic recovery was depressed to a greater extent in SRC-3^−/−^ mice than in WT mice. In addition, the number of BM-nucleated cells in SRC-3^−/−^ mice was significantly reduced and the BM histology of SRC-3^−/−^ mice showed aplasia on the 7th and 14th day following irradiation. To detect the hematopoietic ability of SRC-3^−/−^ mice, the colony-forming ability of granulocytes, erythroid cells and megakaryocytes was investigated. It is clear that hematopoietic stem cells (HSCs) proliferate and differentiate into hematopoietic progenitor cells (HPCs) and lineage-committed HPCs. The count of colony-forming units of hematopoietic cells represents the proliferation ability of hematopoietic stem and progenitor cells (14). It was observed that in SRC-3^−/−^ mice, the count of colony-forming units was significantly decreased on the 7th day and the counts of CFU-E and CDU-Mk were significantly reduced on the 14th day following irradiation, which suggests a high radiation sensitivity of HSCs and HPCs of SRC-3^−/−^ mice. Collectively, the data from the present study indicate a more severe radiation damage to the hematopoietic cells in SRC-3^−/−^ mice.

The SRC-3 gene and protein are widely expressed and exert extensive biological effects on target cells and tissues. SRC-3 acts as a nuclear receptor coactivator and is involved in hormone-regulated functions, including growth and development, sexual maturation and energy metabolism. Furthermore, SRC-3 is a transcriptional coactivator for other transcription factors, including nuclear factor-κB (NF-κB), activator protein-1 (AP-1), E2F transcription factor 1 (E2F1) and insulin-like growth factor I (IGF-I) (15–18). Since SRC-3 was initially reported as an oncogene amplified in breast cancer 1 (AMB1), it has been widely studied as a key cancer regulator rather than a nuclear receptor coactivator (5,9). Accumulating evidence has shown that SRC-3 is involved in initiation, progression and metastasis in numerous cancer types by promoting cell proliferation and resisting apoptosis through both nuclear receptor-dependent and/or -independent pathways (19–21). For example, SRC3 is able to increase cyclin D1 expression and activate the Akt signaling way to promote the proliferation and survival of breast cancer cells (22,23). Depletion of SRC-3 significantly increases the percentage of cells in G1/G0 phase and decreases the percentage of cells in G2/M phase in a mouse model of TR-β-induced thyroid cancer (24). Furthermore, it was found that SRC-3 decreases the expression of the apoptotic inhibitors Bcl-2 and p53 in certain cancer cell lines to evade apotosis (25–26).

Several studies on SRC-3 in the blood cancer cells are available. Colo *et al* (12) reported that SRC-3 was over-expressed in human chronic myeloid leukemia K562 cells and resisted tumor necrosis factor-related apoptosis-inducing ligand (TRAIL)-induced apoptosis. Li *et al* (13) also reported that SRC-3 affected the cell cycle and stimulated cell proliferation by the protein kinase B (AKT) signaling pathway in K562 cells. Another study showed that the downregulation of SRC-3 was involved in deguelin-induced apoptosis in Jurkat cells by NF-κB target gene inhibition (27). These studies confirm that SRC-3 has a role in the proliferation and inhibition of apoptosis in hematopoietic cells. However, its role and mechanism in the hematopoietic system *in vivo* remain unclear. In the present study, the number of hematopoietic cells including blood cells, BM-nucleated cells and colony-forming units was significantly decreased in SRC-3^−/−^ mice compared with WT mice. Radiation-induced hematopoietic injury is in fact an effect on hematopoietic cells. The surviving cells may be able to restore their hematopoietic ability and undergo hematopoietic recovery following irradiation. Guided by previous studies (12,13) on SRC-3, the mechanism of the effect of SRC-3 on hematopoietic cells was investigated through several methods, including cell cycle changes, an increase in the number of cells in G1/G0 phase, surviving cell reserve, stimulation of cell proliferation and resistance to apoptosis. The present study initially validated that SRC-3 protein is expressed in BM-nucleated cells in mice and disruption of SRC-3 may lead to a more severe radiation damage to hematopoietic cells in SRC-3^−/−^ mice compared with those in the WT.

Another noteworthy finding of the present study is a delayed thrombocyte recovery in SRC-3^−/−^ mice. In SRC-3^−/−^ mice, the PLTs recovered less rapidly following irradiation, megakaryocytes were fewer in the BM histological examinations and the number of CFU-Mks was significantly lower until the 14th day following irradiation. These findings suggest an even greater effect of SRC-3 on megakaryocyte lineage than granulocyte and erythroid lineages, which is likely to be attributed to the interaction between SRC-3 and estrogen receptors (ERs). It was identified that ERs were expressed in megakaryocytes (28). Bord *et al* proved that the mRNAs of ERα and ERβ were transcribed and the corresponding proteins were expressed during the differentiation of human megakaryocytes and estrogens, which may markedly stimulate megakaryocyte colonies *in vitro* (29).

In conclusion, the present study showed a more significant decrease in the number of hematopoietic cells and slower hematopoietic recovery in SRC-3^−/−^ mice compared with that in WT mice following a sublethal dose of TBI. The hematopoietic ability is significantly impaired in SRC-3^−/−^ mice. The extensive roles of SRC-3 in cell proliferation and inhibition of apoptosis may be involved in the survival of hematopoietic cells that have suffered radiation damage. However, the complex mechanisms of SRC-3 in hematopoiesis remain to be elucidated, and further intensive study is required to investigate the correlation between SRC-3 and hematopoiesis both *in vitro* and *in vivo*.
